# mitch: multi-contrast pathway enrichment for multi-omics and single-cell profiling data

**DOI:** 10.1186/s12864-020-06856-9

**Published:** 2020-06-29

**Authors:** Antony Kaspi, Mark Ziemann

**Affiliations:** 1 Population Health and Immunity Division, The Walter and Eliza Hall Institute of Medical Research, 1G Royal Parade, Parkville, VIC 3052 Australia; 2 Department of Medical Biology, University of Melbourne, 1G Royal Parade, Parkville, VIC 3052 Australia; 3 School of Life and Environmental Sciences, Deakin University, Geelong, Australia

**Keywords:** Bioconductor package, Differential expression, Gene regulation, Multi-omics, Single-cell profiling, Pathway analysis, Gene set enrichment analysis, Multivariate statistics

## Abstract

**Background:**

Inference of biological pathway activity via gene set enrichment analysis is frequently used in the interpretation of clinical and other omics data. With the proliferation of new omics profiling approaches and ever-growing size of data sets generated, there is a lack of tools available to perform and visualise gene set enrichments in analyses involving multiple contrasts.

**Results:**

To address this, we developed mitch, an R package for multi-contrast gene set enrichment analysis. It uses a rank-MANOVA statistical approach to identify sets of genes that exhibit joint enrichment across multiple contrasts. Its unique visualisation features enable the exploration of enrichments in up to 20 contrasts. We demonstrate the utility of mitch with case studies spanning multi-contrast RNA expression profiling, integrative multi-omics, tool benchmarking and single-cell RNA sequencing. Using simulated data we show that mitch has similar accuracy to state of the art tools for single-contrast enrichment analysis, and superior accuracy in identifying multi-contrast enrichments.

**Conclusion:**

mitch is a versatile tool for rapidly and accurately identifying and visualising gene set enrichments in multi-contrast omics data. Mitch is available from Bioconductor (https://bioconductor.org/packages/mitch).

## Background

Functional enrichment analysis describes the various ways that summarised omics data can be used to infer differential expression (DE) of molecular pathways, or more broadly sets of genes that are functionally linked [1]. Enrichment analysis is increasingly being applied to understand patterns of regulation in diseases and may be useful in better classification of patients into subgroups that could benefit from more specific treatments [2]. Indeed, it is reported that measurement of sets of genes rather than individual genes provides a better ratio of signal to noise and more accurate patient classification [3]**.** Commonly, gene sets are curated to have similar molecular or biological function, or be part of the same biochemical or signaling pathway; but can also be derived from empirical omics and in silico studies.

Most commonly used pathway enrichment analysis methods fall into three categories; over-representation analysis (ORA), functional class sorting (FCS) and pathway topology (PT) methods [1, 4, 5]. Over-representation analysis involved the intersection of genes meeting a prespecified significance and/or fold change threshold with a library of gene sets. Statistically higher or lower enrichment is determined with hypergeometric, Fisher exact or other test. Functional class scoring is different because it uses all detected genes in the calculation of pathway regulation, as it does not involve a significance cutoff. There are several valid approaches to this, but all involve scoring of genes by their differential expression, followed by a statistical test to detect enrichment at the upper and lower extremes of the range. PT methods are similar to FCS methods except they take into consideration additional information about how the genes within a set relate to one another. For example taking into account that biological pathways contain both activators and inhibitors, or that genes in a set are correlated or anticorrelated. PT methods are limited in some cases by a lack of fine-grained pathway knowledge as well as differences in pathway mechanisms in cell types under study [4]. Although PT methods are extremely useful, they are not a focus of this study.

One of the first FCS tools to be described was Gene Set Enrichment Analysis (GSEA). In “preranked” mode, this method summarises DE findings (eg: fold change and/or *p*-value) into a single value and then detects enrichment of gene set members at the extremes of this profile. Statistical significance is established by permuting the profile, quantifying how frequent the detected enrichment is in a randomised profile [6]. Pathway analysis research has since been focused on improving the usability, accuracy and efficiency of tools that analyse single omics data sets. For example, FCS tools (geneSetTest, Roast, CAMERA) have been added to the Limma package, providing a GSEA-like functionality entirely in the R/Bioconductor environment [7]. CAMERA is able to estimate and correct for inter-gene correlation that biases enrichment tests [8]. SetRank adjusts for false positives that arise from overlaps in gene sets [9]. Algorithmic advances included in the FGSEA package have allowed a ~ 50 fold increase in permutation calculation speed in pre-ranked enrichment detection in contrast to GSEA [10] which will be important as gene set databases continue to grow. It has also been shown that ensemble methods of enrichment analysis yield higher accuracy than any individual method alone [11]. Furthermore, there is an emerging interest in tools that calculate pathway expression in individual samples, allowing for granular analysis of variability between samples in large groups (eg: PLAGE, GSVA and ssGSEA) [12–14].

Databases such as Gene Expression Omnibus (GEO) are expanding rapidly [15], enabling comparison of many omics studies at the same time given the appropriate analysis tools. As omics profiling techniques continue to diversify and become more widely used, multi-omics studies are becoming more common. For example, the share of multi-omics data sets, called “Superseries” in GEO has increased, from only 4.6% of series in 2005–2009 to 8.1% in Jan 2016 to Aug 2019. In addition, single-cell profiling has grown explosively in the past 5 years thanks to developments in droplet and nanowell technology, facilitating the deconvolution of cell identities in development and in response to stimuli and disease. These trends highlight a need for tools capable of analysing high-dimensional omics data involving many contrasts, profiling technologies and cell types.

The first approach described for multi-contrast FCS analysis is based upon Hotelling’s T^2^ statistic with two contrasts, and more generally Multiple Analysis of Variance (MANOVA) when considering more than two contrasts. With simulated data, MANOVA test compares favourably with respect to sensitivity and specificity in contrast to other multivariate tests available at the time [16]. An alternative approach, Multi Dimensional Gene Set Analysis (MD-GSA) was later devised, and proposes the use of logistic regression for bidimensional FCS analysis [17]. Although originally intended to analyse multiple contrasts on the same experimental platform, the MANOVA test is equally applicable to pathway level integrative analysis of multi-omics data. For example, joint FCS analysis of ranked proteome and transcriptome data [18]. A MANOVA based FCS test was implemented in the MAVTgsa R package, however it is slow due to the use of a computationally intensive permutation procedure, and lacks visualisation features key to interpreting high-dimensional data [19].

To overcome these limitations, we developed mitch, an R/Bioconductor package that facilitates multi-contrast FCS analysis using a rank-MANOVA approach. We demonstrate the utility of mitch in a variety of use cases including enrichment analysis of multi-omics and single cell transcriptomics. Using simulated data, we benchmark accuracy and execution time of mitch.

## Implementation

### Overview

**Fig. 1:**
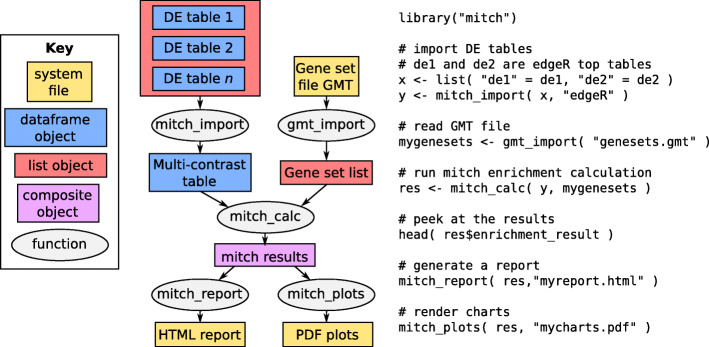
Overview of the mitch workflow. The mitch package consists of five functions (left). The mitch_import function recognises the format of commonly used differential omics tools such as DESeq2, edgeR and limma and performs ranking of each contrast, to create a multi contrast rank table. If user’s would like to use a different ranking scheme, mitch_import can be bypassed in favour of a custom ranking approach. The gmt_import function reads gene matrix transposed files (GMT). The mitch_calc function determines the degree of enrichment of each gene set in the multi contrast table, yielding a mitch result object. The mitch_report function produces a single HTML format report of results containing several tables and charts. The mitch_plots function generates high resolution PDF containing charts derived from the mitch results. Example minimal mitch analysis code (right) to determine the enrichment of gene sets obtained from a GMT file in two dimensions, represented as two edgeR top tables

We provide a schematic diagram and example code to demonstrate a typical mitch workflow from DE tables through to enrichment results (Fig. 1). mitch consists of five functions; mitch_import, gmt_import, mitch_calc, mitch_plots and mitch_report, which are described in the sections below.

### DE scoring and import

To facilitate rank based FCS analysis, DE results for each gene need to be summarised into a single score. mitch_import has the ability to import data from a range of upstream DE packages used in transcriptomics, epigenetics and proteomics (Table 1) [7, 20–43]. Where available, mitch uses the DE test statistic for each gene/protein if available, otherwise calculating the directional significance score (*D*) defined as:

$$ D=-{\log}_{10}\left(\mathrm{nominal}\;p-\mathrm{value}\right)\times \operatorname{sign}\left({\log}_2\mathrm{FC}\right) $$

**Table 1:** mitch can import profiling data generated by a wide range of upstream tools

Target application	Tool	Reference	Function	Ranking metric
RNA-seq (and other applications of count based quantification)	edgeR	[20]	topTable()	“logFC” and “PValue”
	DESeq2	[21]	results()	“stat”
	ABSSeq	[22]	results()	“foldChange” and “pvalue”
	topConfects	[23]	edger_confects() limma_confects()	“confect”
	fishpond/Swish	[24]	swish()	“stat”
	NOIseq	[25]	noiseq()	“ranking”
	Ballgown	[26]	stattest()	“fc” and “pval”
	TCC	[27]	getResult()	“m.value” and “p.value”
	Sleuth	[28]	sleuth_results()	“b” and “pval”
	Cufflinks	[29]	cuffdiff	“test_stat”
Expression microarray	limma	[8]	topTable()	“t”
	DEDS	[30]	topgenes()	“t”
scRNA-seq (and other applications of barcoded cell based count quantification)	Seurat	[31]	FindMarkers()	“avg_logFC” and “p_val”
	Muscat	[32]	pbDS()	“logFC” and “p_val”
	scde	[33]	scde.expression.difference()	“Z”
	MAST	[34]	zlm()	“Coef” and “Pr(>Chisq)”
	DEsingle	[35]	DEtype()	“foldchange” and “pvalue”
Methylation array	missMethyl	[36]	topTable()	“t”
	DMRcate	[37]	extractRanges()	“meanbetafc” and “Stouffer”
Differential proteomics	DEP	[38]	get_results()	“ratio” and “p.val”
	msmsTests	[39]	msms.glm.pois(), msms.glm.qlll() or msms.edgeR()	“LogFC” and “p.value”
	plgem	[40]	plgem.deg()	“PLGEM.STN” and “p.value”
	SDAMS	[41]	SDA()	“beta” and “pv_2part”
	DEqMS	[42]	DEqMS	“t”
Differential binding	DiffBind	[43]	dba.report()	“Fold” and “p.value”

If a different upstream tool is used or if users prefer to use a different DE scoring approach, mitch allows import of “prescored” data. By default, only the genes that are detected in all contrasts are included, but this behaviour can be modified to accommodate sparse datasets such as single cell transcriptomics. During import, users may specify a two-column table that relates gene identifiers in the DE analysis to those in the gene sets. Genes are then ranked from most up-regulated to most down-regulated in each contrast. Gene ranks are centred around the midpoint for each contrast, where the test statistic/directional significance score is zero.

### Gene set definition

A gene set library for use with this implementation must be a named list of character vectors. The gmt_import function reads gene matrix transposed (GMT) system files and is based upon a function originally written for the clusterProfiler package [44] that is interoperable with FGSEA [10]**.**

### Multi-contrast enrichment analysis

The mitch_calc function performs the calculation of multidimensional enrichment and post-hoc univariate enrichments. Only gene sets with 10 or more members present in all contrasts are included by default although the minimum set size threshold can be altered as desired. The base R manova() and summary.manova() functions are used to calculate and report the probability that genes in a set show a multidimensional enrichment as compared to genes not in the set using the Pillai–Bartlett test statistic [45]. The maximum number of contrasts (dimensions) handled by this function is 69. If only one DE profile is provided, then mitch will perform an ANOVA test using the aov() function. The *p*-values are adjusted for multiple comparisons using the false discovery rate (FDR) method of Benjamini and Hochberg [46]. Separately, the enrichment score (*s*) of each gene set is calculated in each contrast as described previously [18].

$$ s=2\ \left({\mathrm{R}}_1-{\mathrm{R}}_2\right)/\mathrm{n} $$

Where R_1_ is the mean rank of genes in the set, R_2_ is the mean rank of genes not in the set and n is the number of genes overall. With two or more contrasts, *S* is defined as the higher dimensional but non-directional enrichment score and is calculated as the Pythagorean distance from the origin.

On Unix based systems, these calculations are distributed on multiple cores to take advantage of multi-threaded computers and save time. End users can prioritise results in three ways; (i) based on statistical significance (low *p*-value), (ii) effect size (large *S*) or (iii) standard deviation (SD) of *s* values across contrasts. SD prioritisation may be of use when searching for gene sets with discordant regulation. End users may also select the number of gene sets for which detailed reports are to be generated downstream; with a default of 50.

### Visualisation of results

The mitch_plots function generates several plots in high resolution PDF. The mitch_report function generates an HTML report with the same outputs, but in a lower resolution to facilitate easy sharing of results. These visualisation functions are limited to 20 or fewer contrasts. Outputs contain scatterplots of DE scores derived from the directional *p*-value method, filled contour plots of ranked profiles, histogram of gene set sizes, scatter plot of effect size measured by *S* distance and statistical significance measured as -log_10_(FDR MANOVA), and a pairs plot of *s* values for all gene sets. In addition, detailed plots are generated for a specified number of gene sets according to the prioritisation approach selected. These include pairwise filled contour plots, pairwise scatter plots and violin plots of enrichments in each contrast. These plots are generated with base R tools or ggplot2 [45, 47]. The HTML output is a self contained report with results tables and charts. Some of these are interactive charts and are generated using the echarts4r package [48].

## Methods

### Case study 1: multi-contrast enrichment analysis of RNA-seq data

RNA-seq data from a previous study with GEO accession GSE109140 [49] were obtained via DEE2 [50]. Transcript-level counts were aggregated to gene level counts using the Tx2gene function of the getDEE2 R package (obtained 2019-10-25). Genes with fewer than 10 reads per sample were excluded from analysis. Two DE contrasts were performed. In contrast 1, normal (5.5 mM) and high (20 mM) glucose were compared. In contrast 2, the effect of 1.0 mM valproic acid (VPA) was assessed in the high glucose condition. DE analysis was performed with DESeq2 v1.22.2 and profiles were imported with mitch. Gene sets used in this study were obtained from Reactome [51]. These and all subsequent numerical analyses were performed in R (v3.6.1) [45].

In order to test whether mitch controls type I errors (false positives) appropriately, three types of randomisation were performed. (i) Shuffle the names of genes in the profile. This retains the correlation structure of the profile dataset. (ii) Shuffle the profile data values. This doesn’t preserve profile correlation structure. (iii) Create random gene sets by sampling gene names from the profile. Gene sets sizes are equal to those obtained from Reactome. The above were repeated 1000 times with the set seed varied between 1 and 1000.

### Case study 2: multi-omics enrichment analysis

Datasets corresponding to A549 (adenocarcinomic human alveolar basal epithelial cell) with and without exposure to 1 h 100 nM dexamethasone were selected to showcase the application of mitch to multi-omics data (listed in Supplementary Table 1) [52]. ChIP-seq and ATAC-seq alignment files in BAM format were downloaded from the ENCODE website. FeatureCounts v1.6.4 [53] was used to count reads mapped to regions within 1 kbp of transcriptional start sites. These coordinates were generated using GTFtools [54] from GENCODE v29 annotations [55]. RNA-seq gene expression counts were downloaded from the ENCODE web site. ChIP-seq, ATAC-seq and RNA-seq underwent differential analysis with DESeq2 v1.22.2 after excluding genes with fewer than 10 reads per sample on average across each experiment. Data were imported with mitch and enrichment analysis was performed with Reactome gene sets as above.

### Case study 3: comparing enrichment results downstream of different DE tools

RNA-seq data from a previous study with GEO accession GSE93236 [56] were obtained via DEE2. Transcript-level counts were aggregated to gene level counts as above. Non-target control and Set7 knock down datasets were compared using different DE tools; DESeq2 (v1.22.2), edgeR glmLRT and QL (v3.24.3), voom-limma (v3.38.3) and ABSSeq (v1.36.0). mitch was used for enrichment analysis using Reactome gene sets. UpSetR package v1.3.3 was used to intersect gene sets that were FDR < 0.05 in each DE tool profile [57]. Pairwise correlation, heatmap, violin and bar charts were generated in R.

### Case study 4: enrichment analysis of single cell sequencing data

Single cell RNA-seq expression data derived from peripheral blood mononuclear cells exposed to interferon beta or vehicle control [58] were obtained, preprocessed and underwent differential state analysis using the “pseudobulk” method as described in the Muscat v0.99.9 vignette [32]. Spearman correlation (*ρ*) of DE values are presented as a heatmap. Mitch was performed with Reactome gene sets and sets with FDR MANOVA < 0.05 were prioritized based on significance, magnitude of *S* and SD of *s*.

### Accuracy of single and dual contrast enrichment detection

In order to establish the accuracy of mitch in comparison to other tools for enrichment analysis, we used a simulated RNA-seq data approach. A human RNA-seq data set with accession number ERR2539161 with 367 M reads assigned to genes was downloaded from DEE2. We simulated a typical RNA-seq experiment with a control/case design with 3 replicates. The starting dataset was downsampled repeatedly to 10 M, 40 M and 100 M reads followed by multiplication by a random noise factor. Noise factors were randomly generated by sampling with the rnorm function with a mean of 2 and a set SD between 0 and 0.6 followed by log_2_ transformation. For enrichment analysis testing, 1000 gene sets were created by randomly sampling 50 gene names. In each simulation 25 randomly selected gene sets were set to be upregulated, and another 25 were set to be downregulated. Fold changes of 2 and − 0.5 were incorporated into the ‘case’ profiles by multiplication of the fold change with the gene counts. If a gene was selected to be both up and downregulated, then no fold change was incorporated. Count matrices then underwent differential analysis with DESeq2 and downstream enrichment analysis with hypergeometric test (phyper, base R v3.6.1), FGSEA (v1.11.1) and mitch. For hypergeometric test, genes with DESeq2 FDR values < 0.05 were included in over-representation analysis. For FGSEA and mitch, the DESeq2 test statistic was used for ranking. In FGSEA, 1000 permutations were performed. Gene sets with FDR values < 0.05 were considered significant and contributed to the calculation of precision, recall and F1 score. Mean results are shown after 500 simulations.

For dual contrast enrichment, data were simulated as above, one control group and two case groups were created to generate two contrasts. FGSEA, mitch and MD-GSA (v1.18.0) were compared.

### Accuracy in detecting multi-contrast enrichment

A random differential expression profile in five dimensions (contrasts) was simulated by repeatedly shuffling ranks for the hyperglycemia data. A library of 1000 gene sets, each with 50 members was created as above and 20 of these gene sets were selected for differential expression. The ranks of the gene set members were shifted based upon a prespecified *s* value using the equation.

$$ {\mathrm{R}}_2-{\mathrm{R}}_1=\mathrm{n}\times s/2 $$

Values for *s* in the five contrasts were generated from a normal distribution with a mean of zero and SD varied between 0 and 0.25. This equates to mean absolute values of *s* between 0 and 0.2. After mitch analysis, a 5% FDR threshold was applied to calculate precision, recall and F1 score. The simulation was repeated 1000 times for each value of SD.

### Execution time

A typical mitch analysis was defined as consisting of a profiling of 20,000 genes with a gene set library of 1000 sets, and each set consisting of 50 members. Gene names, data points and gene sets were randomly generated. Execution time was measured on a 3.8 GHz AMD Ryzen Threadripper 1900 × 8-core (16 thread) processor with 64 GB RAM running Ubuntu 18.04 and R v3.6.1. For comparison, FGSEA was run with 1000 or 2000 permutations. MAVTgsa v1.3 was run with 1000 permutations and MD-GSA using default parameters. Parameters including number of contrasts, number of genes in the profile, number of gene sets and size of gene sets were also varied to determine impact on mitch execution time.

## Results

### Case study 1: multi-contrast enrichment analysis of RNA-seq data

A common use case for mitch is the multi-contrast gene set enrichment analysis of transcriptome data. To demonstrate this, we applied mitch to RNA-seq data initially described by Felisbino et al. [49], consisting of two contrasts; (i) low glucose (LG) versus high glucose (HG); and (ii) HG versus HG with valproic acid (HGVPA). The goal of this study was to identify individual genes and Reactome gene sets dysregulated due to high glucose stimulation in hepatocytes and attenuated with VPA, a clinically prescribed histone deacetylase inhibitor.

**Fig. 2:**
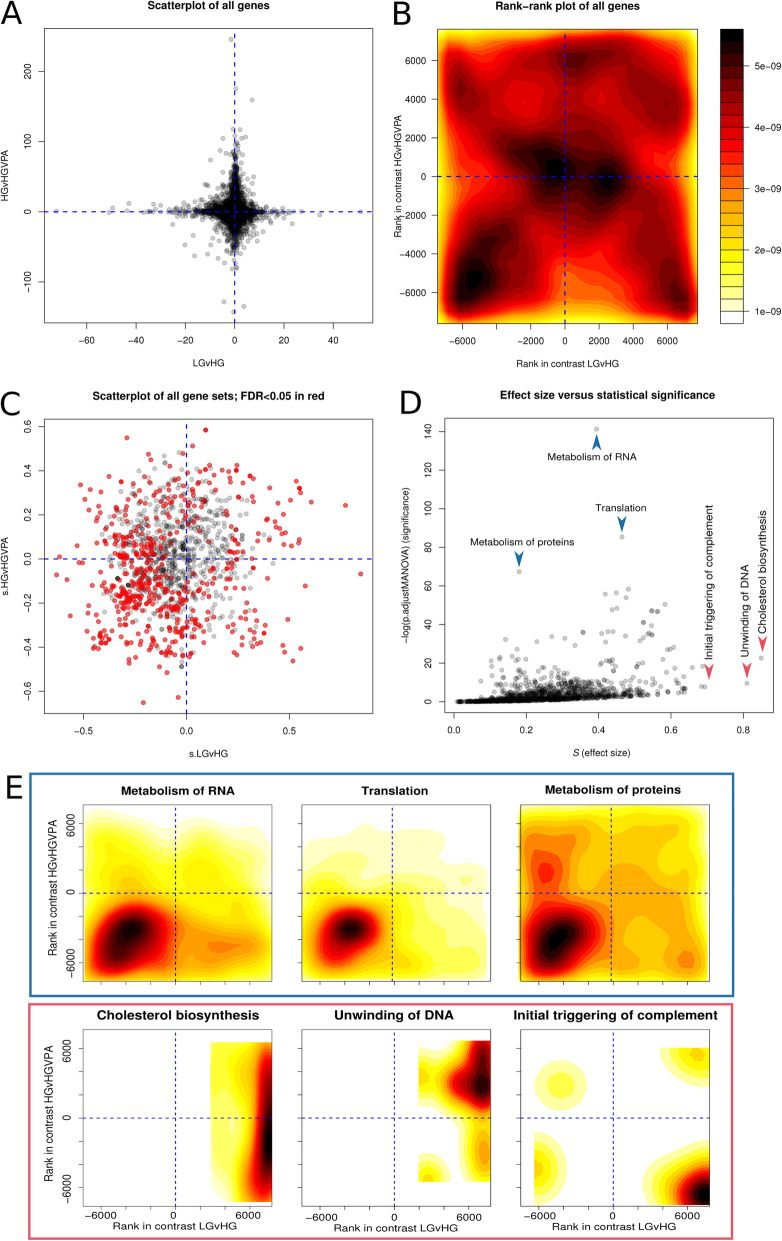
Multi-contrast enrichment analysis of RNA-seq with mitch. **a** The scoring metric, *D*, of every gene in the two contrasts, LG vs HG and HG vs HGVPA. **b** A filled contour plot of all genes after ranking. **c** Enrichment of Reactome gene sets in the two dimensional space. **d** Plot of gene set effect size (*S*) and significance. *S* is defined as the Pythagorean distance from the origin to each point in (**c**). Significance is measured as the -log_10_(FDR MANOVA). **e** Density plots for the three top significant gene sets (blue box) and three gene sets with largest effect size (red box)

There were 15,240 genes with detectable expression in both contrasts. DE scoring with the Wald test statistic provided by DESeq revealed gene expression differences were larger in response to VPA (y-axis) as compared to HG (x-axis) (Fig. 2a), although genes were evenly distributed in all four quadrants (Fig. 2b) and the contrasts were not strongly correlated (Spearman’s *ρ* = 0.010). After exclusion of 967 gene sets with fewer than 10 detected members, 1296 gene sets underwent multi-contrast enrichment analysis with mitch. From the 1296 Reactome gene sets considered, 561 gene sets received FDR MANOVA< 0.05 (Fig. 2c). There were 372 sets with FDR MANOVA< 0.01. A plot of effect size versus statistical significance for each gene set (Fig. 2d) demonstrates the three gene sets with the greatest effect size, while satisfying FDR MANOVA< 0.05, are not highly ranked when prioritising solely on statistical significance. Bidimensional enrichment plots for the top three gene sets based on statistical significance and effect size further show differences in types of associations identified (Fig. 2e). When prioritising by statistical significance, top gene sets are likely to be larger (contain more genes) but more dispersed; while prioritisation by effect size emphasizes smaller gene sets with larger magnitude changes.

**Fig. 3:**
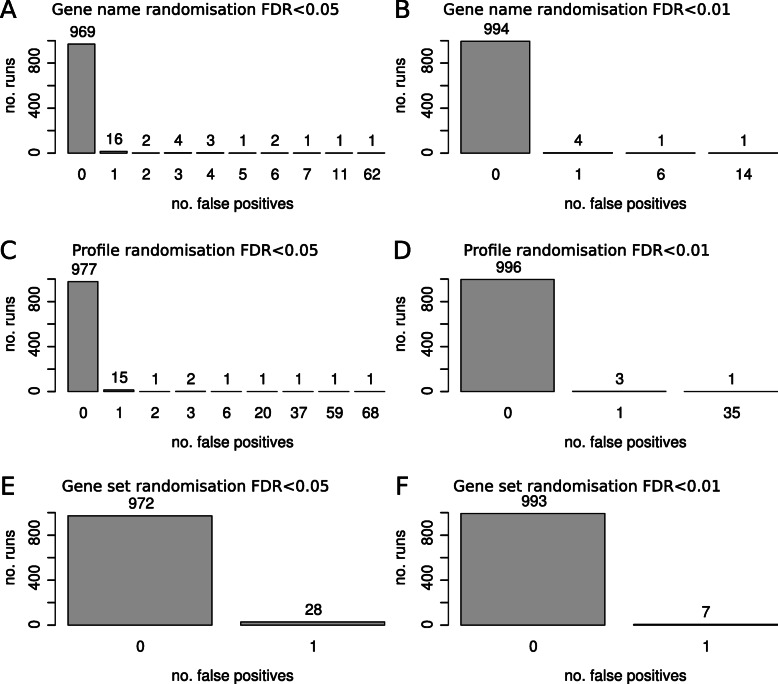
Data randomisation demonstrates robust control of false positives. Data shown in Fig. 2 underwent three types of randomisation. (**a** and **b**) Results of shuffling of gene names in the profile followed by mitch analysis with an FDR threshold of 0.05 and 0.01. **c** and **d** Results of shuffling profile data points, two contrasts shuffled independently. **e** and **f** Randomisation of gene sets by sampling gene names from the profile. All procedures were repeated 1000 times

To demonstrate appropriate control of false positives, three randomisation procedures were performed on bidimensional profiling data shown in Fig. 2. Shuffling gene names 1000 times resulted in an average of 0.141 and 0.024 false positives per run at FDR < 0.05 and FDR < 0.01 respectively (Fig. 3a and b). Shuffling the profile data resulted in an average of 0.213 and 0.038 false positives per run at FDR < 0.05 and FDR < 0.01 respectively (Fig. 3c and d). Randomisation of gene sets resulted in an average of 0.028 and 0.007 false positives per run at FDR < 0.05 and FDR < 0.01 respectively (Fig. 3e and f). Randomisation shows mitch appropriately controls for false positives.

### Case study 2: multi-omic enrichment analysis

**Fig. 4:**
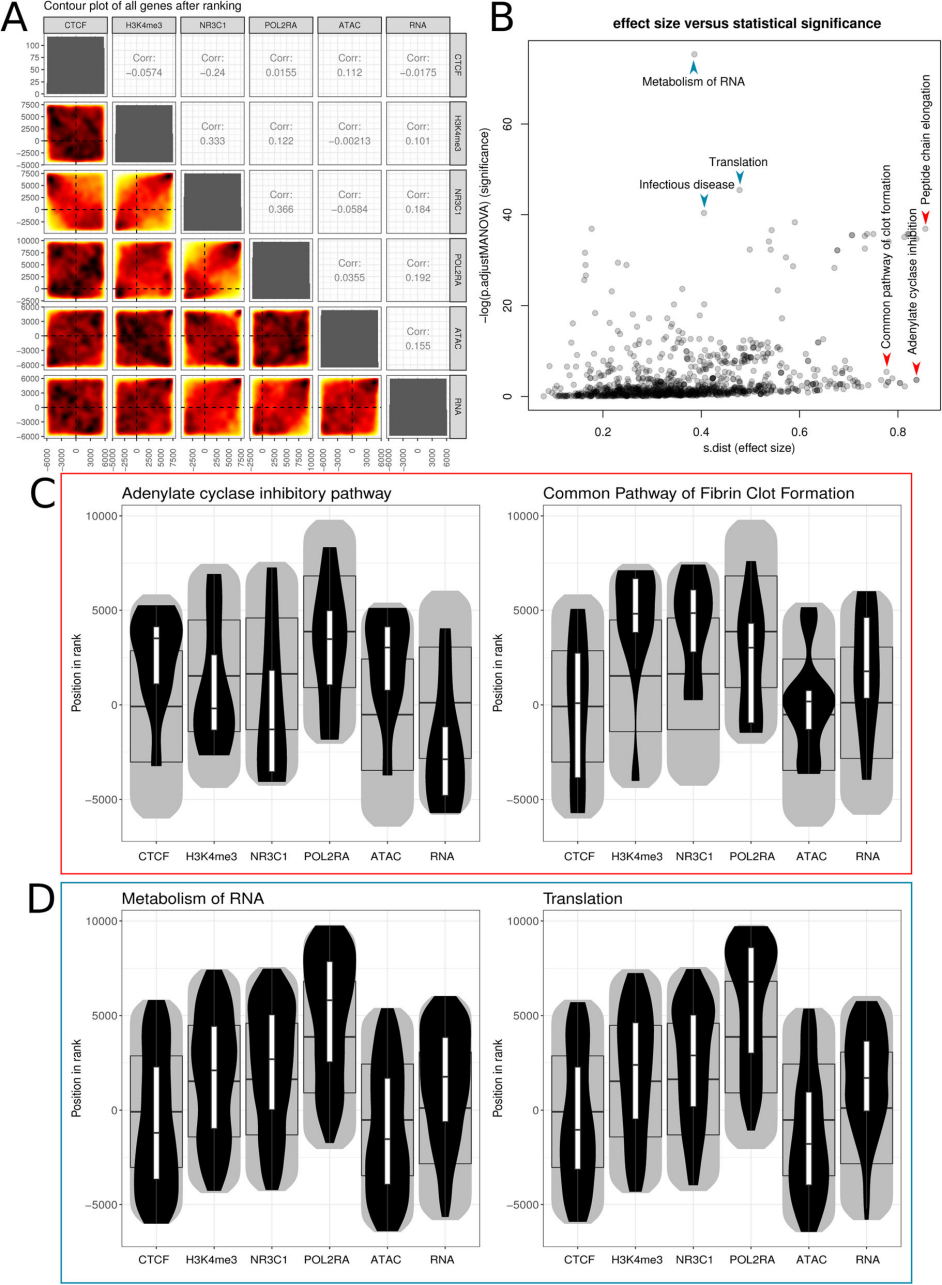
Multi-omics analysis of A549 cells treated with dexamethasone. **a** Pairwise filled contour plots of ranked profiles shows underlying correlations. **b** Plot of gene set effect size and significance. **c** Example gene sets that have large effect sizes. **d** Example gene sets with small FDR values. For **c** and **d**, grey areas denote the distribution of ranks of all detected genes, with median and quartiles depicted by the wide boxplot. Distribution of gene set members is shown by the black violin, with median and interquartile ranges given by the narrow boxplot

Another common use case for mitch is in enrichment analysis of multi-omics data. Previously, the ENCODE consortium have performed multi-omics profiling of dexamethasone (an anti-inflammatory corticosteroid drug) on adenocarcinomic human alveolar basal epithelial cell line A549 [52]. We obtained RNA-seq, ATAC-seq and promoter based ChIP-seq for CTCF, H3K4me3, NR3C1 and POL2RA profiling data for dexamethasone treated and control samples (datasets listed in Supplementary Table 1), followed by differential analysis and then mitch. We found that overall, promoter based NR3C1 occupancy was most positively correlated with POL2RA and negatively correlated with CTCF occupancy. As expected, RNA expression differences were positively correlated with NR3C1, H3K4me3, POL2RA and ATAC-seq signal (Fig. 4a). Selected gene sets with the largest effect size (*S*) include peptide chain elongation, adenylate cyclase inhibition and common pathway of clot formation, while the gene sets with the smallest FDR adjusted *p*-values included metabolism of RNA, translation and infectious disease (Fig. 4b). Adenylate cyclase inhibition genes were associated with increased occupancy of CTCF and chromatin accessibility (inferred from ATAC-seq), but lower RNA expression, H3K4me3 and NR3C1 occupancy (Fig. 4c). Common pathway of fibrin clot formation genes were elevated in H3K4me3, NR3C1, and to a lesser extent in RNA expression. Metabolism of RNA and translation were elevated in POL2RA occupancy and RNA expression (Fig. 4d). Dysregulation of the adenylate cyclase, clot formation pathway and effect on protein synthesis are consistent with the known effects of glucocorticoids [59–62]. This highlights the ability of mitch to identify enrichments in multi-omics datasets, and recover known biology.

### Case study 3: comparing enrichment results downstream of different DE tools

**Fig. 5:**
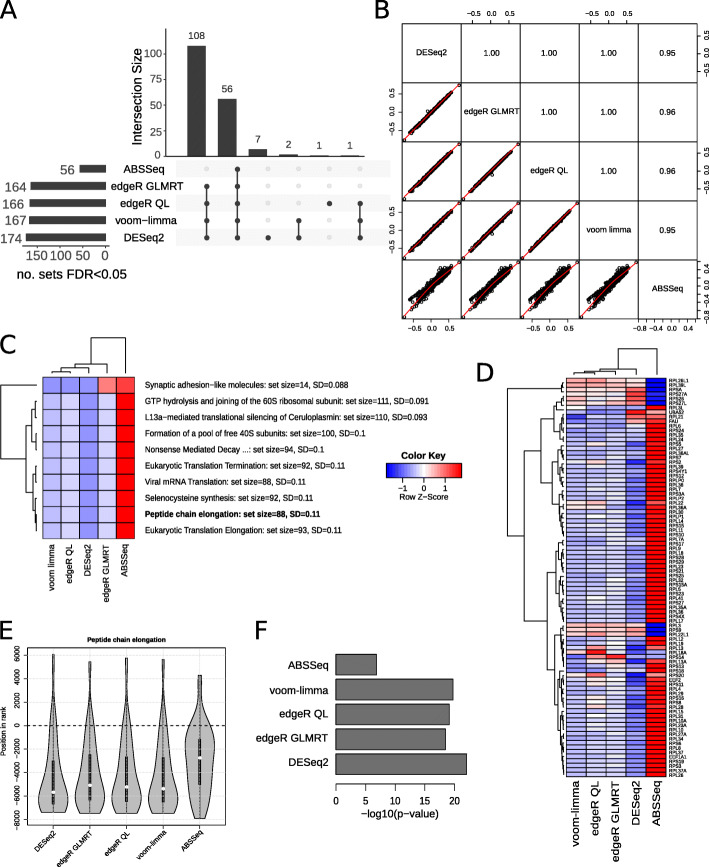
Comparison of gene set enrichment enrichment results downstream of different DE tools on the same RNA-seq dataset. **a** UpSet plot of gene sets produced by different DE tools FDR < 0.05, as calculated by unidimensional mitch. **b** Pairs plot of mitch *s* values for each gene set as processed by different DE tools. Upper triangle shows Pearson’s *r*. **c** A heatmap of 10 gene sets with the highest SD of *s* values across different DE tools, scaled by row. **d** Heatmap of individual gene members of the peptide chain elongation gene set, scaled by row. **e** Violin plot of enrichments of the peptide chain elongation gene set. **f** Observed nominal ANOVA *p*-value of the peptide chain elongation gene set after analysis with different DE tools

When benchmarking an RNA-seq bioinformatic pipeline it is useful to compare the gene set level results of a single RNA-seq contrast analysed with different DE tools, to determine what effect tool selection has on final results. To demonstrate this, bulk RNA-seq data corresponding to Set7 knock-down and non-target control samples [56] was processed using DESeq2, edgeR glmLRT, edgeR QL, ABSSeq and voom-limma followed by mitch analysis with Reactome gene sets. After DE analysis, there were variable numbers of DE genes at the 5% FDR cutoff (DESeq2: 5150, edgeR glmLRT: 5721, edgeR QL: 5910, voom-limma: 5903 and ABSSeq: 2253). After mitch analysis also with a 5% FDR cutoff, there were variable numbers of differentially regulated gene sets, with ABSseq showing the fewest (Fig. 5a). Only 56 gene sets were common to all DE tools, but the majority (108) were common to all except ABSSeq. A pairs plot of gene set *s* values comparing data from each tool shows the results of DESeq2, edgeR glmLRT, edgeR QL and voom-limma are virtually identical (Pearson *r* > 0.99), while results from ABSSeq are somewhat different (Pearson *r* ~ 0.95) (Fig. 5b). Sorting gene sets by SD of *s* values reveals several gene sets that exhibit stronger downregulation in DESeq2, edgeR glmLRT, edgeR QL and voom-limma as compared to ABSSeq (Fig. 5c). The peptide chain elongation gene set is a prime example, where the majority of genes are downregulated when analysed with DESeq2, edgeR glmLRT, edgeR QL and voom-limma, but appear unchanged when analysed with ABSSeq (Fig. 5d). This difference in collective regulation by ABSSeq is clear when the gene set is visualised as a violin plot (Fig. 5e). As a consequence, the statistical significance of this gene set is lower for ABSSeq compared to the other DE tools (Fig. 5f). These results are generally consistent with previous findings that show ABSSeq is more conservative than other differential RNA-seq tools [22]. This result highlights that choice of DE tools subtly impacts enrichment results and these can be explored using mitch.

### Case study 4: enrichment analysis of single cell sequencing data

**Fig. 6:**
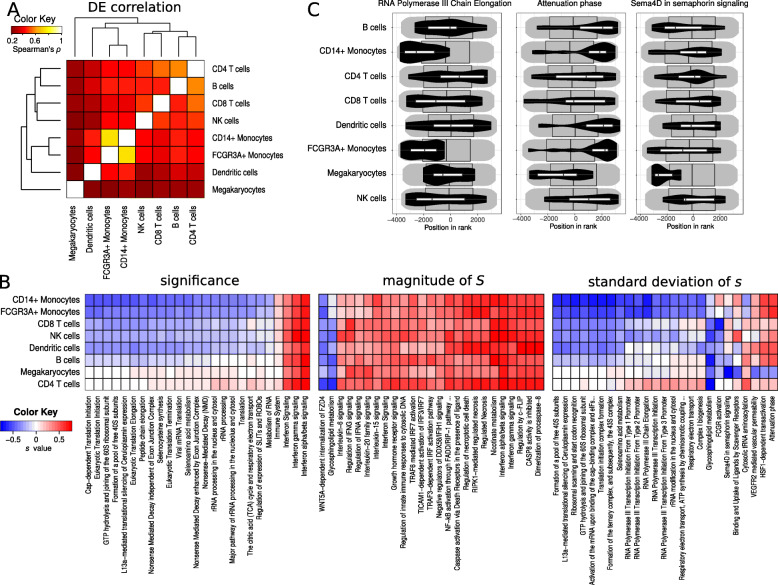
Applying mitch to perform enrichment analysis of scRNA-seq data. **a** Heatmap of Spearman correlation values for DE scores for each cell identity. **b** Heatmaps of mitch *s* scores for top 25 Reactome gene sets after prioritisation by significance, magnitude of *S* and SD of *s.* All gene sets shown are FDR MANOVA < 0.05. **c** Example enrichment plots for three gene sets with high SD of *s* values

Single cell RNA sequencing (scRNA-seq) allows the parallel profiling of hundreds to thousands of individual cells in a sample. As in standard bulk RNA-seq, contrasts between experimental conditions can be made, with the major difference that scRNA-seq provides information on cell identity (also known as “cell type”). Generally, scRNA-seq DE tools provide either test statistic or fold change and *p*-value information for each gene of each cell identity. Here, mitch can be applied to perform enrichment analysis by considering the DE profiles of each cell identity as an independent contrast. In order to demonstrate this, scRNA-seq data derived from peripheral blood mononuclear cells exposed to interferon beta or vehicle control as described by Kang et al [58] underwent clustering and differential analysis with Muscat [30] to yield “pseudobulk” DE tables for each cell identity. After scoring DE values, correlation analysis identified Spearman’s *ρ* between 0.23 and 0.57 between cell identities, with lymphocytes grouped together, dendritic cells grouped with monocytes, and megakaryocytes appearing as an outgroup (Fig. 6a).

Again, mitch analysis was performed with Reactome gene sets. Of the 2263 gene sets present, 1629 were excluded due to the detection of fewer than 10 members. Of the 607 sets remaining, 77 were differentially regulated (FDR MANOVA< 0.05). Next, we prioritised the results three ways; (i) significance, (ii) magnitude of *S*, and (iii) SD of *s* values (Fig. 6b). When prioritising by significance, interferon signaling was observed to be upregulated as expected, but there were many housekeeping gene sets that were observed to be downregulated, including TCA cycle, rRNA processing and translation. When prioritising by magnitude of *S*, there was a larger number of upregulated gene sets involved in immune responses observed. In general, these gene sets demonstrated coordinated regulation in response to interferon beta stimulation that was consistent between cell identities. The value of scRNA-seq over bulk is the ability to detect cell identities responding differently to a stimulus, so it may be useful to prioritise by the observed SD of *s* across cell identities. Using this approach, we identified several gene sets with discordant cell identity responses to interferon beta, that would be impossible to detect with bulk sequencing (Fig. 6c). For example “RNA polymerase III chain elongation” was downregulated in monocytes specifically, “Attenuation phase” was upregulated in monocytes and B cells but not in megakaryocytes, and “Sema4D in semaphorin signaling” was downregulated in megakaryocytes specifically. This result highlights the utility of mitch in analysing single cell profiling data and the impact of different prioritisation schemes.

### Accuracy of single and dual contrast enrichment detection

**Fig. 7:**
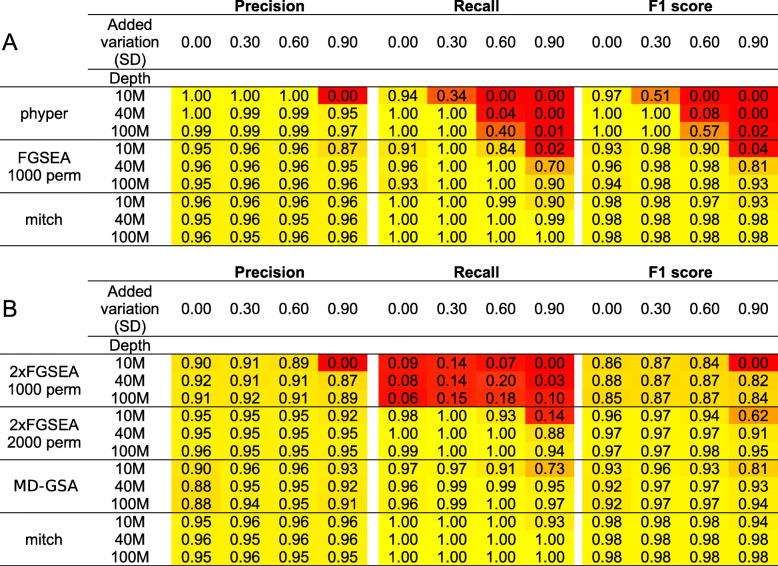
Precision, recall and F1 values for enrichment analysis of simulated RNA-seq datasets. Simulations are based on *n* = 3 control and case replicates with different sequencing depth (10, 40 and 100 million reads) and different degrees of added variation (SD). The mean of 500 simulations is shown. **a** Evaluation of single contrast enrichment. **b** Evaluation of dual contrast enrichment

To test the accuracy of mitch to detect single-contrast enrichments, we undertook a simulation study. Our goal was to determine the performance of mitch and other enrichment tests (FGSEA and hypergeometric test) over a range of typical RNA-seq conditions by varying the sequencing depth and degree of inter-sample variation. We simulated DE to 5% of randomly generated gene sets with equal numbers of sets up and down-regulated (see Methods). Members of those gene sets were given log_2_ fold changes of 1 and − 1 to simulate expression changes. Count matrices underwent DE analysis and gene set enrichment testing with a 5% FDR threshold to calculate precision and recall of these tools, calculated by comparing ground truth values to the observed results (Fig. 7a). As expected, DE results from DESeq2 yielded smaller *p*-values when sequencing depth was greater and inter-sample variation was smaller. This resulted in overall better precision and recall of gene set enrichment results in simulations involving greater sequencing depth and smaller inter-sample variation. In tests with low variance, the hypergeometric test was the most precise, however higher variance caused a severe reduction in recall. In contrast, FGSEA and mitch were more robust to higher variance especially with higher sequencing depth. When variance was low, the accuracy of mitch was similar to FGSEA, but with higher variance, mitch showed superior recall. Potentially, FGSEA’s recall could be improved by using a greater number of permutations.

Next, we applied this approach to the problem of identifying enrichments in two contrasts. We planned to compare the accuracy of FGSEA (run twice), with mitch, mdgsa and MAVTgsa, but thoroughly evaluating MAVTgsa was impractical due to the long computational time (Fig. 9b). We found that FGSEA recall was lower than expected, but this was improved by increasing the number of permutations to 2000. Averaged over the 9 different conditions, mitch demonstrated the highest precision (0.956), recall (0.994) and F1 score (0.974) as compared to the other tools (Fig. 7b). These results demonstrate that mitch has slightly better accuracy than existing tools for single and dual contrast enrichment analysis.

### Accuracy of multi-contrast gene set enrichment detection

**Fig. 8:**
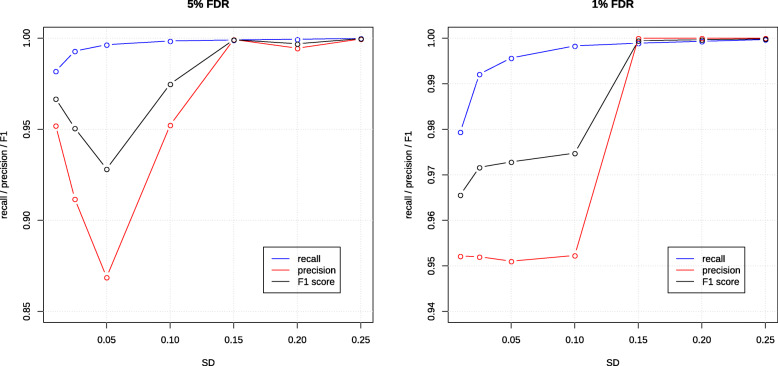
mitch accuracy in distinguishing DE gene sets in a simulated five-contrast dataset. The *x*-axis shows the set SD for sampling *s* values for each gene set for each contrast. *x*-axis can be interpreted as increasing enrichment strength. Values shown are the mean of 1000 replications

Next, we sought to quantify the accuracy of mitch in distinguishing DE gene sets in a simulated five-contrast dataset. Member genes of 20 gene sets were shifted by precomputed *s* values sampled from a range of values with a mean of zero and SD varied from 0 to 0.25 (Methods). After mitch analysis with a 5 and 1% FDR cutoff, precision, recall and F1-score were calculated (Fig. 8). When SD = 0, ie in completely random data, no false positive DE gene sets were found after 1000 replications. As expected, recall increased with larger SD values. False positives showed a non linear relationship with SD. In the 5% FDR trial, precision showed a minimum of 86% when SD values were set to 0.05, however at a more strict 1% FDR cutoff, the minimum precision value was 95%. F1 scores indicated high accuracy with SD values above 0.15, which corresponds to mean absolute *s* values of 0.08 or higher. To put this into context of a real dataset, from the 318 gene sets in case study 2 with FDR < 0.05, there were 12.6% with mean absolute *s* values lower than 0.08 (Supplementary Figure 1). For trials with SD set to ≥0.15, F1 scores were 0.9991 or higher. These findings support the accuracy of mitch in identifying DE gene sets in multidimensional data.

### Execution time

**Fig. 9:**
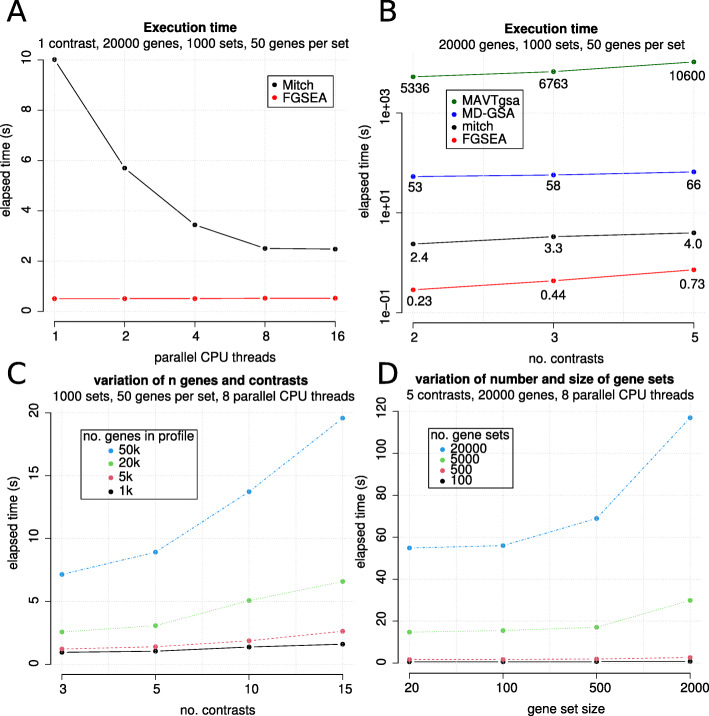
Benchmarking mitch execution time. **a** single-contrast enrichment analysis with mitch and FGSEA (1000 permutations) on up to 16 CPU threads. **b** Comparison of mitch, MAVTgsa, MD-GSA and FGSEA execution times with two to five contrasts. **c** Effect of number of contrasts and genes in the profiling data. (D) Effect of gene set size and number of sets

As execution time is a consideration in big data applications, we measured the execution times of mitch in typical applications. Initially, single-contrast enrichment analysis was tested. A random profile consisting of 20,000 genes and queried with a gene set library of 1000 sets, and each set consisting of 50 members. On a single CPU thread, single-contrast mitch analysis was completed in 10 s; but using 8 threads this was reduced to 2.5 s. Using an additional 8 threads did not speed up execution further (Fig. 9a). FGSEA which is known for its speed, completed the analysis in 0.51 s using the default 1000 permutations with a single thread. Next, the speed of mitch was compared to MAVTgsa, MD-GSA and FGSEA for dual contrast enrichment. We found mitch was 16–22 times faster than MD-GSA and 2000–2500 times faster than MAVTgsa, although not as fast as running FGSEA twice (Fig. 9b). Next, the effect of increasing the number of genes profiled and the number of contrasts on mitch execution time was assessed. The number of genes in the profiling data had a linear effect on mitch execution time, but adding extra contrasts gave a sub-linear increase in execution time (Fig. 9c). When the number and size of gene sets was manipulated, we found the number of gene sets gave a linear increase in mitch execution time whereas an increase in the size of gene sets gave a sub-linear increase in execution time (Fig. 9d). This result indicates that although mitch is slower than FGSEA for single contrast analysis, mitch enables large-scale enrichment analyses within a reasonable time.

## Discussion

Previously, we have used the concept of rank MANOVA enrichment and visualisation in several studies of cardiovascular disease, pharmacology, aging and neurological disease **(eg:** [63, 64]**)**, but only recently has the software become available as a package for wider use. In the process of packaging the software, we have added additional features that will enhance its utility. We have made mitch interoperable with many popular upstream analysis tools, especially those from the Bioconductor community [65]. We have made use of the many and varied visualisation features available in the R environment including filled contour plots, heatmaps, violin plots and taken advantage of interactive charts made possible with HTML embedded JavaScript bindings provided by the echarts4r package [48].

Although mitch was initially developed to compare gene expression signatures in a multi-contrast RNA-seq experiment, it has applications beyond this. Mitch is ideally suited to multi-omics data, as demonstrated in case study 2 above that takes advantage of ENCODE profiling data to identify pathway-level regulatory events associated with dexamethasone exposure. In case study 3, we evaluated the impact of DE tool selection on enrichment results, but this approach could equally be applied to choices of other upstream data processing steps such as choice of mapping, quality control and normalisation methods.

Perhaps the most exciting application for mitch is in the burgeoning field of single cell biology as in case study 4. After data clustering by cell identity and differential state analysis, this type of data can undergo set enrichment analysis. Although unidimensional enrichment tools such as GSEA are already being applied to scRNA-seq data, there are some limitations. The MANOVA approach of mitch is better able to detect enrichments that are subtle but consistent across profiles. Moreover mitch natively summarises the results of its multi-contrast analysis, which means less work for the end user. The different prioritisation modes allows users to focus on findings that are statistically robust, associated with large effect sizes or discordant among cell identities.

Single contrast enrichment simulations show that mitch is as accurate as FGSEA, and that both these methods have better performance over a wider range of input data than the hypergeometric over-representation test (Fig. 7a); similar findings have been noted previously [66]. Randomisation analysis of dual-contrast data shows that mitch yields very few false positives (Fig. 3). In dual-contrast analysis, mitch accuracy is superior to MD-GSA and FGSEA, although FGSEA accuracy could potentially be improved by using more permutations (Fig. 7b).

While in this paper we have limited our analyses here to human biological pathways curated by Reactome, mitch is capable of using gene sets from any source and organism for which such sets are available (eg: [67]). In summary, the functionality provided by mitch makes it a versatile and powerful tool for rapidly distilling pathway level information from large omics datasets.

## Availability and requirements

Project name: mitch.

Project home page: http://bioconductor.org/packages/mitch

Operating system(s): Linux, MacOS and Windows.

Programming language: R.

Other requirements: R v4.0, Bioconductor 3.11.

License: Creative Commons Attribution-ShareAlike 4.0 International Public License.

Any restrictions to use by non-academics: None.

## Supplementary information

**Additional file 1: Supplementary Figure 1.** Mean absolute s scores in case study 2. Results of the multi-omics analysis shown in Fig. 4 underwent filtering based on a 5% FDR threshold before calculating the mean absolute s values in the six-dimensional analysis (left). Next, the number of gene sets with mean absolute s values greater or lower than 0.08 was counted (right) as below this level, mitch precision is lower than expected.

**Additional file 2: Supplementary Table 1.** Multi-omics data derived from control and dexamethasone treated A549 cells obtained from the ENCODE Project web page.

## Data Availability

The mitch package is available from Bioconductor (https://bioconductor.org/packages/mitch). The code used to generate the results shown here has been deposited to GitHub as well (https://github.com/markziemann/mitch_paper). Publicly available data used in this study is available from the respective data banks as outlined in the methods section.

## Abbreviations

BAM: Binary alignment map
DE: Differential expression
DEE2: Digital Expression Explorer2
FDR: False discovery rate
FGSEA: Fast gene set enrichment analysis
GEO: Gene Expression Omnibus
GMT: Gene matrix transposed
GSEA: Gene Set Enrichment Analysis
HG: High glucose
HTML: Hypertext markup language
LG: Low glucose
MANOVA: Multivariate analysis of variance
PDF: Portable document format
SD: Standard deviation
VPA: Valproic acid
