# Supplementary material for: mitch: multi-contrast pathway enrichment for multi-omics and single-cell profiling data
Source: BMC Genomics. 2020 Jun 29;21:447. doi: 10.1186/s12864-020-06856-9 (PMC7325150; doi:10.1186/s12864-020-06856-9)
Supplement: Supplementary file 1 — Additional file 1: Supplementary Figure 1. Mean absolute s scores in case study 2. Results of the multi-omics analysis shown in Fig. 4 underwent filtering based on a 5% FDR threshold before calculating the mean absolute s values in the six-dimensional analysis (left). Next, the number of gene sets with mean absolute s values greater or lower than 0.08 was counted (right) as below this level, mitch precision is lower than expected. [file 12864_2020_6856_MOESM1_ESM.pdf]

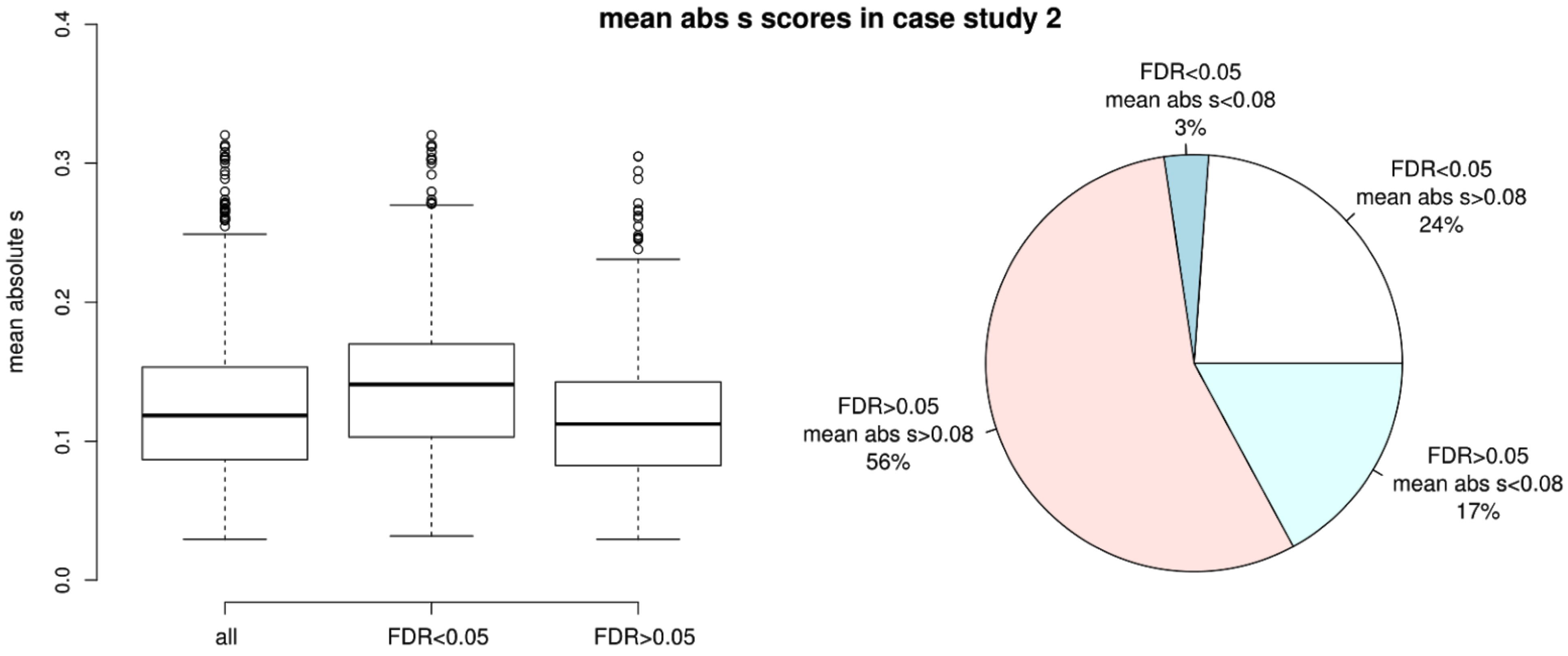

Supplementary Figure 1. Mean absolute *s* scores in case study 2. Results of the multi-omics analysis shown in Figure 3 underwent filtering based on a 5% FDR threshold before calculating the mean absolute *s* values in the six-dimensional analysis (left). Next, the number of gene sets with mean absolute *s* values greater or lower than 0.08 was counted (right) as below this level, mitch precision is lower than expected.
