# Supplementary material for: mitch: multi-contrast pathway enrichment for multi-omics and single-cell profiling data
Source: BMC Genomics. 2020 Jun 29;21:447. doi: 10.1186/s12864-020-06856-9 (PMC7325150; doi:10.1186/s12864-020-06856-9)
Supplement: Supplementary file 2 — Additional file 2: Supplementary Table 1. Multi-omics data derived from control and dexamethasone treated A549 cells obtained from the ENCODE Project web page. [file 12864_2020_6856_MOESM2_ESM.docx]

**Supplementary Table 1: Multi-omics data derived from control and dexamethasone treated A549 cells obtained from the ENCODE Project web page.**

| **Data type** | **File** | **Treatment group** |
| --- | --- | --- |
| ATAC: bam | ENCFF597SLV.bam  ENCFF248HDS.bam  ENCFF978DQZ.bam  ENCFF020COS.bam  ENCFF758ORC.bam  ENCFF809EKV.bam | Dex 100 nM 1 hr  Dex 100 nM 1 hr  Dex 100 nM 1 hr  Ctrl  Ctrl  Ctrl |
| ChIP-Seq for POL2RA: bam | ENCFF380ZHO.bam  ENCFF960AWO.bam  ENCFF471JEL.bam  ENCFF593TPG.bam  ENCFF984VJJ.bam  ENCFF697JDL.bam  ENCFF223EHQ.bam  ENCFF565TWZ.bam | Dex 100 nM 1 hr  Dex 100 nM 1 hr  Dex 100 nM 1 hr  Dex 100 nM 1 hr  Ctrl  Ctrl  Ctrl  Ctrl |
| ChIP-Seq for H3K4me3: bam | ENCFF626NXS.bam  ENCFF978RET.bam  ENCFF945WGW.bam  ENCFF973TUQ.bam  ENCFF428UWO.bam  ENCFF643FMK.bam | Dex 100 nM 1 hr  Dex 100 nM 1 hr  Dex 100 nM 1 hr  Ctrl  Ctrl  Ctrl |
| ChIP-Seq for NR3C1: bam | ENCFF807YIG.bam  ENCFF331QXR.bam  ENCFF038DBH.bam  ENCFF668EHX.bam  ENCFF496BTD.bam  ENCFF681JBZ.bam  ENCFF181HLP.bam  ENCFF870WJP.bam | Dex 100 nM 1 hr  Dex 100 nM 1 hr  Dex 100 nM 1 hr  Ctrl  Ctrl  Ctrl  Ctrl  Ctrl |
| ChIP-Seq for CTCF: bam | ENCFF356NSD.bam  ENCFF951KQC.bam  ENCFF180EPT.bam  ENCFF774IBY.bam  ENCFF713UMA.bam  ENCFF810IXF.bam | Dex 100 nM 1 hr  Dex 100 nM 1 hr  Dex 100 nM 1 hr  Ctrl  Ctrl  Ctrl |
| RNA-seq: Featurecounts tabulated gene expression counts | ENCFF082ICE.tsv  ENCFF467SQA.tsv  ENCFF620TAH.tsv  ENCFF778BJF.tsv  ENCFF744NRK.tsv  ENCFF054BTH.tsv  ENCFF130DRZ.tsv  ENCFF165EET.tsv | Ctrl  Ctrl  Ctrl  Ctrl  Dex 100 nM 1 hr  Dex 100 nM 1 hr  Dex 100 nM 1 hr  Dex 100 nM 1 hr |
